# Recurrent Cervical Lymphadenitis Responding to Antibiotics Turned Out to Be Lymphoma on Biopsy: A Case Report

**DOI:** 10.7759/cureus.62864

**Published:** 2024-06-21

**Authors:** Indrika Sharma, Pankaj Gharde

**Affiliations:** 1 General Surgery, Jawaharlal Nehru Medical College, Datta Meghe Institute of Higher Education and Research, Wardha, IND

**Keywords:** lymph node fnac, recurrent lymph node, hodgkin's lymphoma non-hodgkin's lymphoma, lymph node excision, cervical lymph node metastasis, cervical lymph node, cervical lymph node biopsy, cervical lymphadenitis

## Abstract

Recurrent cervical lymphadenitis is a common clinical presentation often managed with empiric antibiotic therapy. However, despite antibiotic treatment, persistent lymphadenopathy warrants consideration of alternative etiologies, including malignancy. We present the case of a 71-year-old female with recurrent cervical lymphadenitis that initially responded to antibiotics but was ultimately diagnosed as lymphoma upon biopsy. Despite conservative management, the patient's symptoms persisted, prompting surgical excision of the lymph node. Histopathological examination confirmed the lymphoma diagnosis, highlighting the importance of considering malignancy in cases of persistent lymphadenitis. This case underscores the significance of prompt evaluation, including biopsy, to ensure timely diagnosis and appropriate management in patients with recurrent cervical lymphadenitis.

## Introduction

Recurrent cervical lymphadenitis is a common presentation in clinical practice, often attributed to infectious etiologies such as bacterial or viral infections [1]. It is characterized by the repeated inflammation of cervical lymph nodes, resulting in swelling, tenderness, and sometimes systemic symptoms such as fever [2]. While most cases of cervical lymphadenitis resolve with conservative management and antibiotic therapy, persistent or recurrent symptoms may necessitate further investigation to rule out underlying pathology. Lymphoma, a heterogeneous group of lymphoid malignancies, can occasionally present with cervical lymphadenitis, mimicking benign inflammatory conditions [3]. Lymphomas are classified into two main categories: Hodgkin lymphoma (HL) and non-Hodgkin lymphoma (NHL), each with distinct clinical and histopathological features [4]. HL is characterized by the presence of Reed-Sternberg cells within an inflammatory background, whereas NHL comprises a diverse group of lymphoid malignancies with varying histological subtypes [5]. Prompt diagnosis and appropriate management of lymphoma are crucial for optimal patient outcomes, highlighting the importance of considering malignancy in cases of persistent lymphadenitis.

While imaging modalities, such as ultrasound, computed tomography (CT), and magnetic resonance imaging (MRI), can aid in the evaluation of cervical lymphadenopathy, histopathological examination remains the gold standard for diagnosing lymphoma [6]. Fine-needle aspiration cytology (FNAC) and core needle biopsy are commonly utilized techniques for obtaining tissue samples from enlarged lymph nodes, providing valuable diagnostic information [7]. Given the potential for serious underlying pathology, clinicians must maintain a high index of suspicion for malignancy in cases of recurrent cervical lymphadenitis that do not respond to conservative management. Timely referral to a multidisciplinary team, including hematologists, oncologists, and pathologists, is essential for accurate diagnosis and appropriate management of patients with suspected lymphoma.

## Case presentation

**Figure 1:**
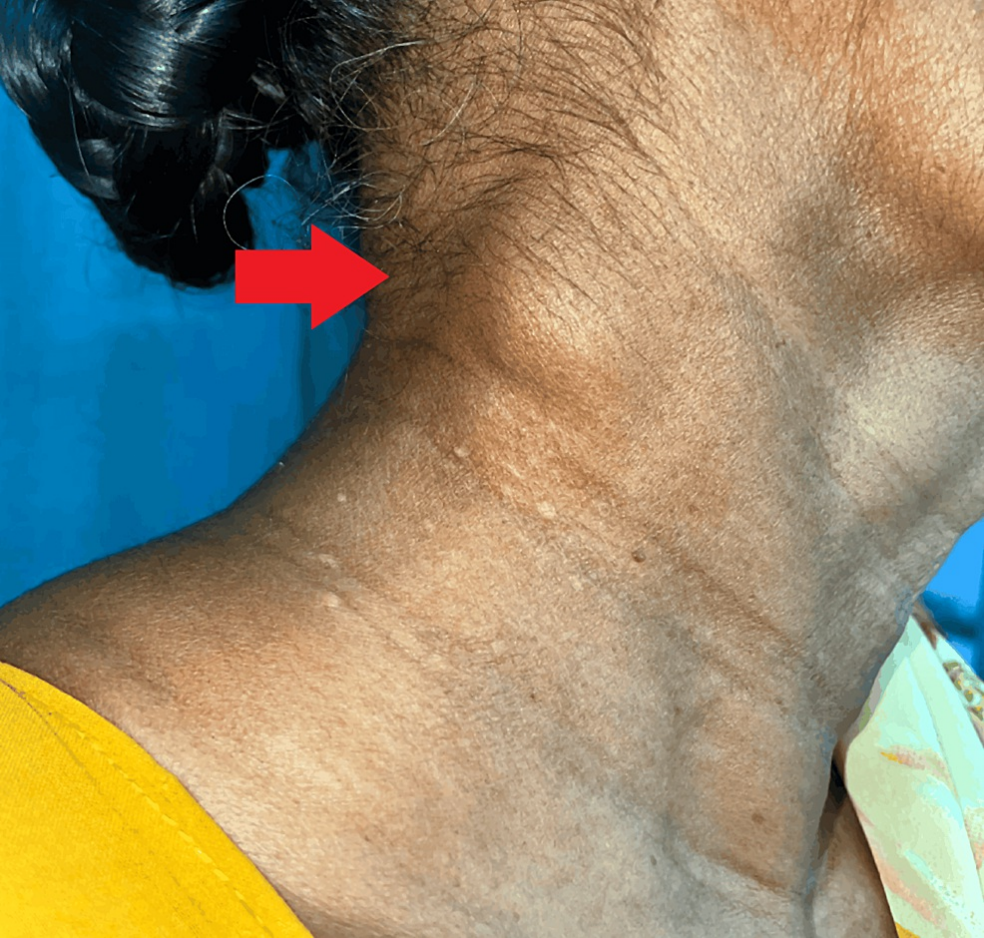
Clinical picture of cervical lymph node

A 71-year-old female presented with a chief complaint of a progressively enlarging swelling over the right posterior triangle of her neck. The swelling initially appeared two months ago and was measured at 1 × 1 cm at onset, gradually increasing to its current size of 2 × 2 cm (Figure 1). The patient described the onset as insidious, and she noted associated pain of a pin-pricking nature, which occasionally radiated. Although she reported experiencing intermittent fever, it was not documented, and she denied any other significant symptoms.

**Figure 2:**
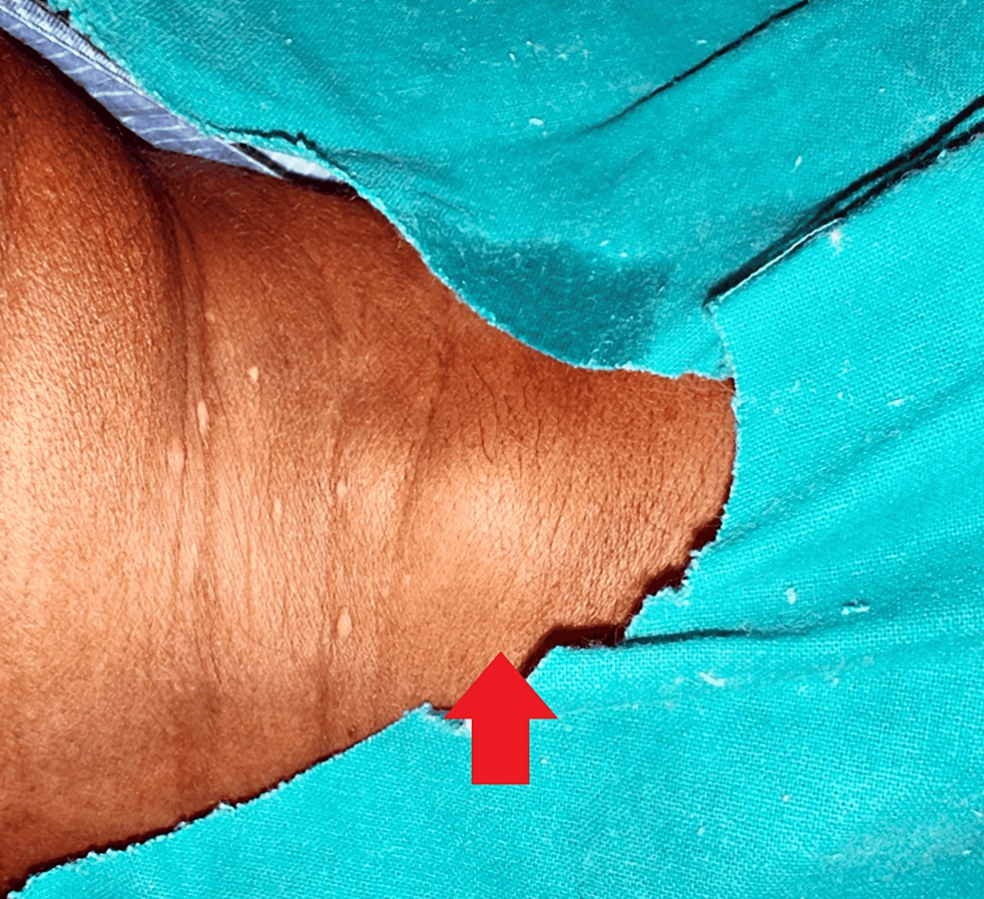
One enlarged lymph node with one small accompanying node

The patient had sought medical attention previously and had been treated conservatively for the swelling without resolution. She had no significant medical history, including no known history of diabetes mellitus, tuberculosis, hypertension, or bronchial asthma. On examination, inspection revealed an erythematous swelling over the right posterior triangle of the neck, approximately 3 cm below the angle of the mandible. The surrounding structures appeared normal, with no engorged veins or dilated vessels noted. Palpation confirmed tenderness and a local rise in temperature over the swelling, which measured approximately 2 × 2 cm in size. She received antibiotics for the same (Tab Augmentin 625 mg PO three times daily (TDS)) for which there was a response and symptoms subsided (Figure 2). The surface was smooth, and the consistency ranged from soft to firm. No fluctuation, pulsation, or translucency was noted.

**Figure 3:**
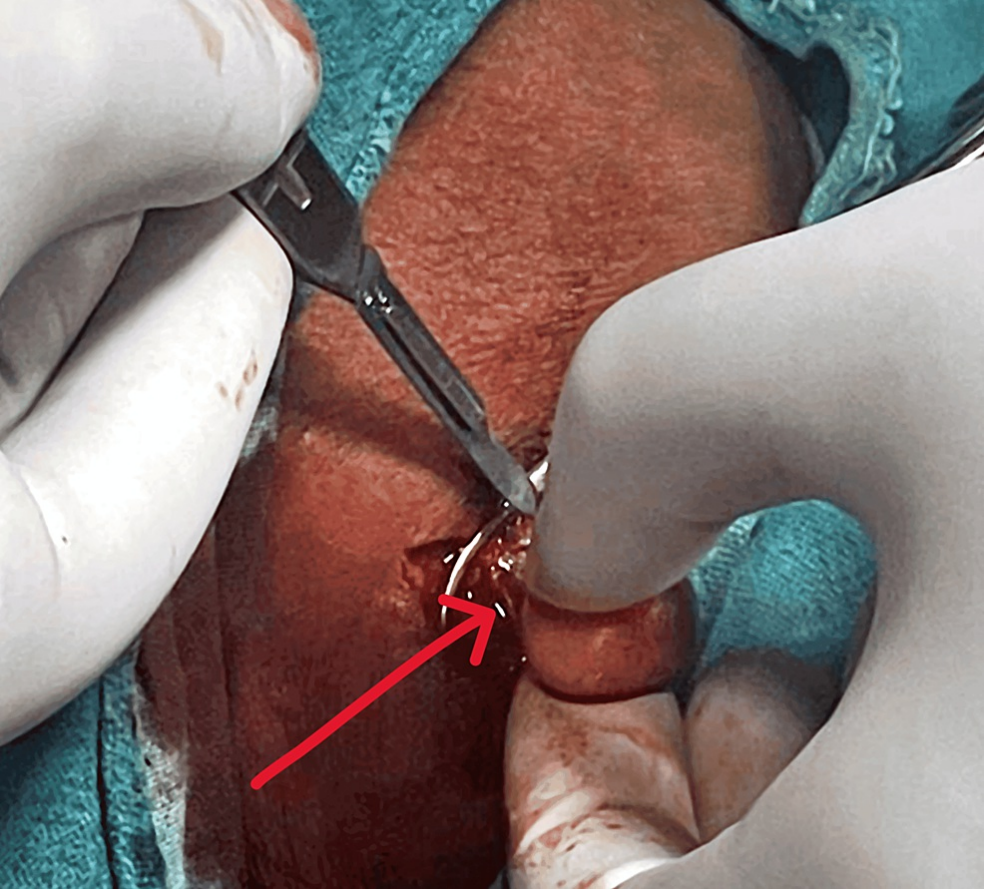
Excision of the cervical lymph node

**Figure 4:**
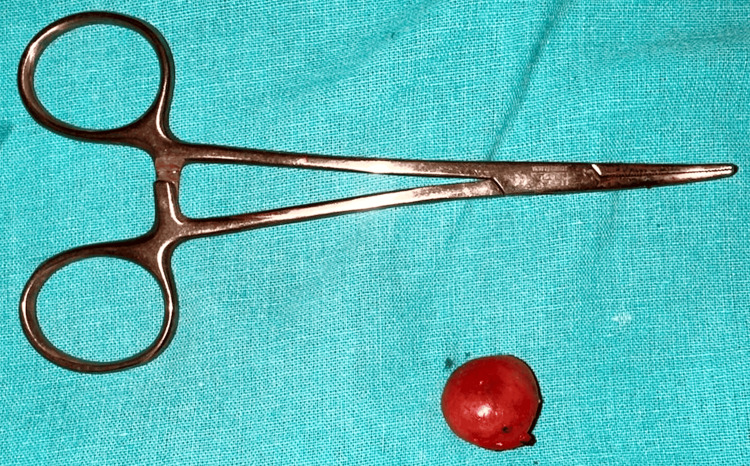
Excised specimen of cervical lymph node

Due to the persistence of symptoms and the inconclusive nature of imaging studies, the decision was made to proceed with surgical excision of the lymph node under general anesthesia. The procedure was performed under all aseptic precautions, with the patient positioned supine. A vertical incision of 3 cm was made over the right cervical region, directly over the lymph node. The incision was deepened until the deep fascia was reached, and the lymph node, measuring 2 × 2 cm (Figure 3), was carefully separated and excised en bloc. Another smaller lymph node measuring 1 × 1 cm was also excised (Figure 4). Following excision, the cavity was thoroughly cleaned with betadine and hydrogen peroxide, and hemostasis was achieved. The subcutaneous layer was closed using Vicryl 3.0 round body (RB), and the skin was closed using Ethilon 3.0 reverse cutting (RC). A sterile dressing was applied, and the patient was transferred to the postoperative ICU for observation.

**Figure 5:**
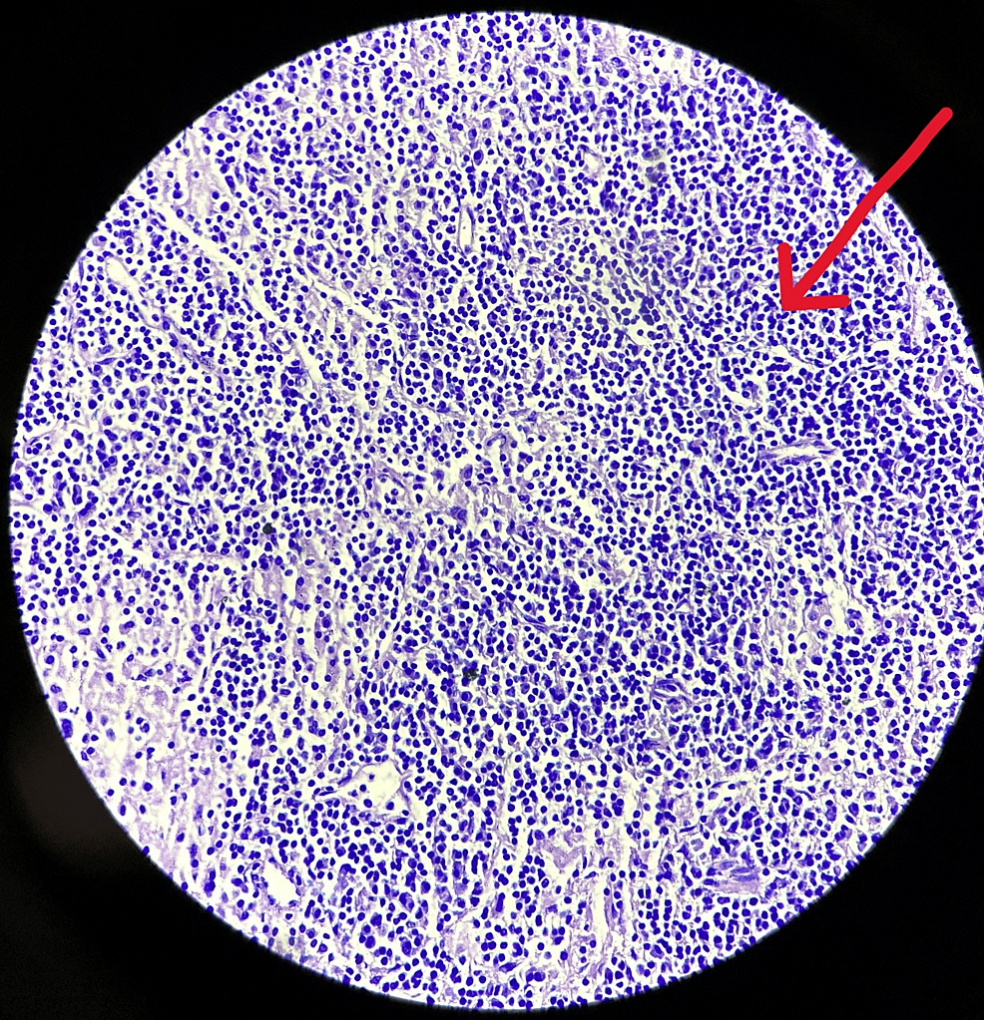
The microscopic image of Hodgkin lymphoma A peripheral blood smear was taken and examined under the microscope, showing a bilobed to multilobed nucleus, prominent eosinophilic nucleolus creating lacunae-like spaces, pale retracted cytoplasm, and mixed inflammatory background.

A histopathological examination of the excised lymph node was awaited to confirm the diagnosis of HL (Figure 5). Despite the absence of immediate complications, close postoperative monitoring was warranted, and the patient was advised to follow up in the surgery outpatient department upon discharge. Further management would be guided by the histopathology report and the patient's clinical status, considering the possibility of underlying malignancy. Given the patient's age, thorough evaluation and a multidisciplinary approach would be crucial in determining the appropriate action.

## Discussion

Recurrent cervical lymphadenitis is a common clinical presentation that often prompts empiric treatment with antibiotics. However, when symptoms persist despite antibiotic therapy, clinicians must consider other potential etiologies, including malignancy. Our case report underscores the importance of maintaining a high index of suspicion for malignancy in cases of recurrent lymphadenitis, particularly in older patients. Lymphadenopathy in the cervical region can result from various causes, including infections, autoimmune diseases, and malignancies [8]. While infectious etiologies, such as bacterial or viral infections, are the most common causes of lymphadenitis, lymphoma must be considered, especially in persistent or progressive lymphadenopathy [9]. In our case, the patient's symptoms failed to resolve with conservative management and antibiotic therapy, prompting further investigation.

Surgical excision and histopathological examination remain the gold standard for diagnosing lymphoma. In our case, surgical excision of the enlarged lymph node was performed under general anesthesia to obtain a definitive diagnosis. Histopathological examination revealed the presence of lymphoma, confirming the suspicion of malignancy. This highlights the crucial role of biopsy in cases where malignancy is suspected, as clinical and radiological findings alone may not provide a definitive diagnosis. The diagnosis of lymphoma in our patient underscores the importance of considering malignancy in the differential diagnosis of persistent lymphadenopathy, particularly in older individuals. While lymphoma can occur at any age, the incidence increases with advancing age, with most cases diagnosed in individuals over 60 years old [10]. Therefore, age should be factored into the evaluation of lymphadenopathy, and thorough investigation is warranted in older patients presenting with persistent lymphadenitis.

Management of lymphoma requires a multidisciplinary approach involving hematologists, oncologists, and surgeons. Treatment options for lymphoma vary depending on the subtype and stage of the disease but may include chemotherapy, radiation therapy, immunotherapy, or stem cell transplantation [11]. In our case, the patient's management plan was determined based on the histopathology report, and further treatment decisions were made in consultation with hematology/oncology specialists.

## Conclusions

In conclusion, our case report emphasizes the critical need for vigilance and thorough evaluation in cases of recurrent cervical lymphadenitis, particularly in older individuals. Despite initial response to antibiotic therapy, persistent or progressive lymphadenopathy should prompt consideration of underlying malignancy. Surgical excision and histopathological examination remain pivotal in achieving a definitive diagnosis, as highlighted in our case where lymphoma was ultimately identified. This underscores the importance of a multidisciplinary approach involving clinicians, surgeons, and pathologists to ensure accurate diagnosis and timely initiation of appropriate management. Moving forward, clinicians should maintain a high index of suspicion for malignancy in cases of persistent lymphadenitis, especially in older patients, to optimize patient outcomes through early intervention and tailored treatment strategies.
